# The role of Caveolin-1 in tumor-derived extracellular vesicle biology and its implications

**DOI:** 10.3389/fcell.2025.1656953

**Published:** 2025-09-02

**Authors:** Satish Kailasam Mani, Christophe Lamaze, Cristian Saquel

**Affiliations:** ^1^ Membrane Mechanics and Dynamics of Intracellular Signaling Laboratory, Institut Curie - Centre de Recherche, PSL Research University, Paris, France; ^2^ Institut National de la Santé et de la Recherche Médicale (INSERM), U1339, Paris, France; ^3^ Centre National de la Recherche Scientifique (CNRS), UMR 3666, Paris, France

**Keywords:** caveolae, extracellular vesicles, cancer, mechanics, metastasis

## Abstract

Tumor-derived extracellular vesicles (TEVs) are increasingly recognized as key mediators of intercellular communication between cancer cells and their environment, a process crucial for tumor progression. TEVs can act locally on neighboring cells or travel long distances to impact remote tissues, thereby promoting tumor growth, cell invasion, pre-metastatic niche formation, and ultimately, metastasis. Despite significant insights into the molecular mechanisms by which TEVs shape the tumor microenvironment (TME) and induce pro-metastatic effects in recipient cells, many questions remain unanswered. Recent studies suggest that caveolae, invaginations of the plasma membrane with critical roles in cellular mechanics, may play an important role in TEV-mediated metastatic trait acquisition by cancer cells. The presence of caveolin-1 (Cav1) in EVs supports its involvement in EV dynamics, including biogenesis, secretion and uptake by recipient cells. Further research into the role of Cav1 in EV-mediated cancer progression could pave the way for improved diagnostic tools and novel therapeutic strategies in cancer treatment.

## 1 Introduction

Cancer remains the second leading cause of mortality worldwide, accounting for nearly nine million deaths per year (WHO). The cancerous process starts with the acquisition of hallmark features, primarily genetic mutations in cells, which may be initiated by carcinogen exposure or inherited. These mutations alter the normal functions of the genes responsible for regulating cell growth, division, and other essential cellular processes, disrupting the balance between cell proliferation and cell death. Uncontrolled cell division and rapid proliferation of mutated cells mark the initial stage of cancer, resulting in the formation of a primary tumor. As cancer progresses, some cells break away from the primary tumor in a process known as metastasis, enabling them to colonize neighboring tissues and organs. These cancer cells migrate through blood or lymphatic vessels, eventually establishing secondary tumors in distant parts of the body (Castaneda et al., 2022). The unique ability of cancer cells to metastasize represents one of the greatest challenges in cancer treatment and significantly impacts patient prognosis. Understanding the different stages of cancer progression is essential for developing early detection methods, effective treatments, and improving recovery outcomes for patients.

Tumor extracellular vesicles (TEVs) have emerged as key players in multiple stages of cancer progression. Extracellular vesicles (EVs) are small, membrane-bound particles released by various cell types, offering valuable insight into the intricate mechanisms driving cancer progression. TEVs act as intercellular messengers, delivering selective cargo capable of influencing tumor growth, metastasis, immune escape, and treatment resistance (Kalluri and McAndrews, 2023).

In this review, we delve into the role of tumor-derived EVs in cancer progression. We describe how these nanoscopic messengers contribute to tumor growth and dissemination and highlight their potential as biomarkers for early diagnosis and as therapeutic targets. Furthermore, we shed light on the involvement of the caveolar protein caveolin-1 in EV biology, including its role in EV biogenesis, uptake, and impact on recipient cells from a cancer-specific perspective.

## 2 Extracellular vesicles

In 1967, Peter Wolf and, in 1968, H. Clarke Anderson published the first electron micrographs of EVs, revealing the presence of small particles outside the cell. They referred to these particles as platelet dust and matrix vesicles, respectively (Anderson, 1969; Wolf, 1967). Following this discovery, extensive research was conducted to characterize the functionality of these vesicles. For the next 3 decades however, EVs were largely considered a cellular disposal system, primarily responsible for discarding unwanted material (Johnstone et al., 1991). This simplistic view changed drastically in 1996 with the first functional description of EVs produced from immune cells. EVs were found to carry MHC class II molecules, endowing them with the capacity to present antigens to immune cells and activate immune responses (Raposo et al., 1996). This seminal study highlighted the functional significance of EVs in various biological processes, including their role in pathological conditions. This study sparked a renewed interest in EV research, leading to a wealth of information about the diverse subtypes of EVs, their composition, cargo, and their physiological roles in both health and disease.

### 2.1 EV subtypes

EVs are highly heterogeneous, and their classification remains a sensitive and evolving subject requiring careful consideration. Historically, EVs were categorized based on their size, with small EVs measuring less than 200 nm and large EVs exceeding 200 nm. Another widely used classification relies on their biogenesis pathway. Exosomes are EVs that originate intracellularly from a specialized subset of endosomes, known as multivesicular bodies (MVBs), which contain intraluminal vesicles (ILVs). The fusion of MVBs with the plasma membrane (PM) allows the release of exosomes into the extracellular milieu. In contrast, ectosomes are formed by outward budding directly from the PM. Additionally, specific subpopulations of EVs are produced during the onset of distinct cellular processes, including apoptotic bodies, migrasomes, protrusion-derived EVs, and midbody remnants (van Niel et al., 2018). The Minimal Information for Studies of Extracellular Vesicles (MISEV) 2023 guidelines recommend using the generic term “EV” along with operational descriptors (e.g., 100 k g pellet) rather than relying on terms like “exosomes” and “ectosomes”. These traditional terms suggest a specific origin of biogenesis, which can be inconsistently defined and potentially misleading unless the subcellular origin is definitively demonstrated (Théry et al., 2018).

### 2.2 EV biogenesis

The biogenesis of EVs can be broadly categorized according to the dependence or not on the endosomal sorting complexes required for transport (ESCRT) protein machinery. The ESCRT-dependent pathway begins with the recognition and recruitment of ubiquitinated cargo proteins by the ESCRT-0 complex, composed of two subunits, HRS and STAM, which contain ubiquitin-binding domains. These proteins facilitate cargo clustering at the early endosomal membrane (Migliano et al., 2022; Raiborg and Stenmark, 2009). Next, ESCRT-I is recruited by ESCRT-0 (Hurley and Hanson, 2010), further clustering the ubiquitinated cargo and acting as a bridge between ESCRT-0 and ESCRT-II (Carlton and Martin-Serrano, 2007; Katzmann et al., 2001). ESCRT-II, which also contains ubiquitin-binding domains, stabilizes the ESCRT-III complex, responsible for membrane constriction and scission (Alam et al., 2004). The ESCRT-III complex drives membrane scission and ILV formation while recruiting accessory proteins that catalyze the disassembly of the complex (Hurley and Hanson, 2010; Wollert et al., 2009).

MVBs can also form independently of ESCRT proteins. Ceramide, a bioactive lipid, can drive the formation of lipid microdomains with spherical membrane curvature, leading to inward budding and ILV formation (Trajkovic et al., 2008). Another pathway in MVB biogenesis involves tetraspanins, such as CD63, CD81, and CD9, which are protein scaffolds embedded in EV membranes and involved in ILV cargo sorting and biogenesis (Guix et al., 2017; van Niel et al., 2011).

The biogenesis of ectosomes share similarities with exosome formation, including the involvement of the ESCRT machinery and tetraspanins for smaller ectosomes that precipitate at the same speed as exosomes. However, ectosomes vary in size (100 nm to over 1 μm), and their biogenesis mechanisms are more diverse. The formation of large ectosomes remains poorly understood but is thought to involve actin cytoskeleton rearrangements, membrane blebbing, and subsequent fission of the bleb (Di Vizio et al., 2009).

The composition of EVs often mirrors the physiological and pathological state of their parental cells. EV cargo can reflect external stimuli, such as nutrient availability, oxygen levels, and physical cues, as well as internal changes, including altered metabolism, autophagy, senescence, and oxidative stress (Dixson et al., 2023). EVs transport a wide array of biologically active molecules, including metabolites, proteins, lipids, genetic material (e.g., RNA and DNA). Protein cargo is typically loaded into EVs by the ESCRT machinery, while RNA-binding proteins assist in RNA loading. The lipid composition of EVs varies depending on their subcellular origin (Lee et al., 2024).

### 2.3 EV secretion and uptake

In the case of MVBs, the successful secretion of exosomes involves additional machineries beyond the ESCRT complexes. The fusion of endosomes with the PM and the subsequent release of exosomes occurs through a multistep GTPase-switching process. This involves the sequential endosomal recruitment of small GTPases, including Rab7, followed by Arl8b and Rab27, before fusion with the PM (Verweij et al., 2022). SNARE proteins also play a critical role in mediating MVB-PM fusion. Specifically, syntaxin-4, SNAP-23, and VAMP-7 are essential for this process, as their deletion impairs MVB fusion and EV secretion (Liu et al., 2023).

The biological outcomes of EV-mediated communication between cells largely depend on the efficient uptake of EVs by recipient cells. This complex process involves multiple pathways and sequential steps, starting with EV docking at the PM (Russell et al., 2019). Tetraspanins, which are abundant in EVs, interact with various receptors on recipient cells, facilitating EV binding, uptake, and targeted delivery to specific cells or tissues (Morelli et al., 2004; Rana et al., 2012). Additionally, integrins present on EVs and recipient cells contribute EV-PM binding. Inhibition of integrin binding can disrupt EV adhesion and subsequent uptake (Altei et al., 2020; Hoshino et al., 2015).

After docking at the PM, EVs can deliver their cargo through two primary mechanisms. First, EVs may directly fuse with the recipient cell membrane, merging their lipid bilayers and releasing their contents into the cytoplasm. This fusion process relies on interactions between membrane outer leaflets and the participation of fusogenic proteins, including SNAREs and Rab proteins, located on both EVs and recipient cell membranes. Second, EVs can be internalized via various endocytic pathways, leading to their encapsulation in endosomes. Once inside endosomes, EVs may fuse with the endosomal membrane to release their cargo into the cytoplasm (Mulcahy et al., 2014). Several endocytic pathways can be used when direct fusion does not occur. These include phagocytosis, macropinocytosis, clathrin-mediated endocytosis, and Cav1/caveolae-mediated endocytosis (Delenclos et al., 2017). Each pathway contributes to the cellular uptake of EVs, highlighting the complexity and adaptability of EV-mediated intercellular communication. The role of these endocytic pathways in the selective activity of internalized EVs remains poorly understood.

### 2.4 EVs in cancer

In cancer, both direct and indirect communication between healthy and tumor cells play pivotal roles in determining cancer progression. Indirect communication mediated by EVs has been implicated in multiple aspects of oncogenesis, tumor progression and metastasis.

**FIGURE 1:**
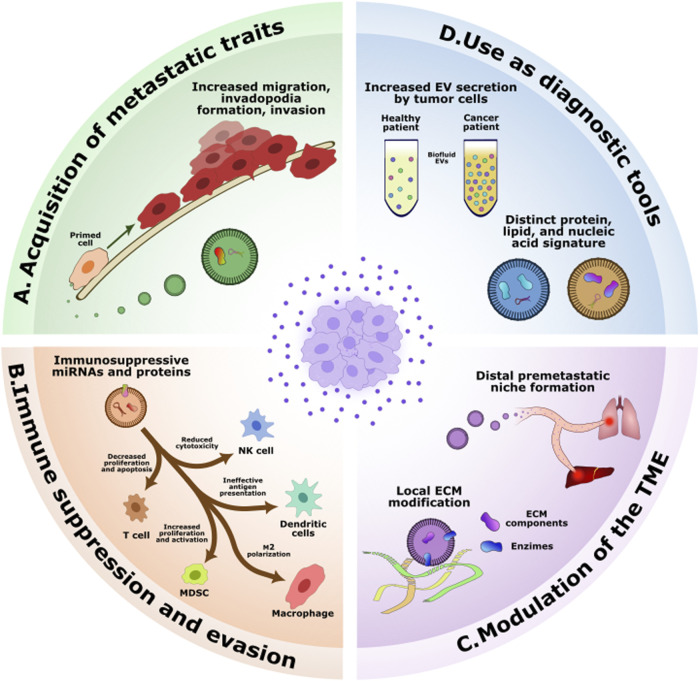
EVs in cancer. Schematic representation of the challenges and opportunities of EV in cancer. **(A)** Cancer patients exhibit increased levels of EVs in biofluids such as blood, plasma and cerebrospinal fluid, with a distinct cargo from healthy EVs. **(B)** EVs are able to modulate the tumor microenvironment locally by modifying their ECM and distally by travelling to other organs and establishing pre-metastatic niches. **(C)** EVs can exert an immunosuppressive effect on immune cells by blocking their functions or inducing apoptosis. **(D)** Tumor EVs are able to confer metastatic traits to non-cancerous cells, including increased migration, invasion and invadopodia formation.

Oncogenic mutations in healthy cells, a hallmark of cancer initiation, can alter the secretion patterns and cargo composition of released TEVs. These EVs often carry oncogenic molecules such as proteins and miRNAs that promote proliferation in recipient cells or suppress anti-tumor responses (Bebelman et al., 2018). In both autocrine and paracrine manners, TEVs can activate signaling pathways that sustain proliferation, enable evasion from apoptosis, and enhance metastatic phenotypes (Semeradtova et al., 2025; Wang et al., 2020). Upon uptake by recipient cells, TEVs can drive invasive behaviors, including increased migration, invasion and invadopodia formation (Figure 1, panel A) (Liguori and Kralj-Iglič, 2023).

TEVs also play a crucial role in immune evasion. Tumor cells release TEVs carrying immunosuppressive molecules such as PD-L1, FasL, Trail, and immunoregulatory miRNAs. These components suppress the activity of immune cell, including T cells, dendritic cells, MDSCs, macrophages, and natural killer cells, impairing the body’s ability to mount an effective anti-tumor response (Figure 1, panel B) (Cai et al., 2019; Lopatina et al., 2022; Wei et al., 2023).

Additionally, TEVs modulate the tumor microenvironment (TME) by transporting extracellular matrix (ECM) components and ECM modifying enzymes. These cargoes reshape the TME, promote angiogenesis, activate survival signaling pathways, and stimulate migration and invasion of cancer cells (Nawaz et al., 2018; Patel et al., 2024). Beyond local remodeling, TEVs can also prepare distant organs for metastasis by establishing pre-metastatic niches, making them more susceptible to colonization by migrating cancer cells. This selective organotropism may explain why certain cancer types preferentially metastasize to specific organs (Figure 1, panel C) (Ghoroghi et al., 2021; Nguyen et al., 2022).

TEVs also hold great promise as non-invasive diagnostic and prognostic tools. They carry protein, DNA and RNA biomarkers that reflect the state of their parental tumor cells. TEVs can be detected in bodily fluids, making them accessible through liquid biopsy techniques, such as mass spectrometry, enabling non-invasive detection and characterization. Their specific biomarkers provide valuable insights into the presence, progression, and treatment response of various cancers, thereby facilitating early detection, monitoring, and the possibility of tailored therapeutic strategies (Figure 1, panel D) (Hinestrosa et al., 2022).

Interestingly, the sheer quantity of EVs released by cancer cells can itself serve as an indicator of oncogenic activity. Cancer cells are known to release EVs in significantly higher quantities than healthy cells, increasing their concentration in bodily fluids like plasma (Figure 1, panel D) (Kharmate et al., 2016; Logozzi et al., 2009; Riches et al., 2014). This increased secretion reflects the abnormal cellular environment of tumors, driven by factors such as mechanical stress from dense TME packing, metabolic starvation, autophagy, acidic pH, and hypoxic conditions (Logozzi et al., 2009; Patwardhan et al., 2021; Wang X. et al., 2023; Wang Z. et al., 2019).

In the broader context of TEV’s role in cancer progression, caveolin-1 (Cav1) the primary component of caveolae, has emerged as a key focus of interest. Cav1 is involved in EV biogenesis, cargo selection, and the pro-tumorigenic effects mediated by TEVs. However, the precise mechanisms by which Cav1 contributes to cancer progression through TEVs remain to be fully elucidated.

## 3 Caveolae

### 3.1 Structure and composition

Caveolae are small, typically 50–100 nm in diameter, cup-shaped invaginations visible on the cytoplasmic face of the plasma membrane through electron microscopy. Present in most cell types, they play essential roles in cellular processes such as signal transduction, lipid regulation, and mechanical response. Caveolae are highly enriched in cholesterol, sphingolipids, and glycosphingolipids, which contribute to their structural integrity and functional properties (Del Pozo et al., 2021; Lamaze et al., 2017; Parton, 2018). Structurally, caveolae are characterized by a protein coat primarily composed of caveolins (caveolin-1, caveolin-2, caveolin-3) and cavins (cavin-1, cavin-2, cavin-3, cavin-4). Among these, caveolin-1 (Cav1) and cavin-1 are indispensable for caveolae biogenesis. Cav1 serves as a scaffold that binds cholesterol and lipids while cavin proteins form a coat complex on the cytoplasmic surface, stabilizing the structure and contributing to membrane curvature (Kozlov and Taraska, 2023; Parton et al., 2021). Additional accessory proteins, including members of the EHD and pacsin families, contribute to caveolae stability and dynamics (Ludwig et al., 2013; Ludwig et al., 2016; Morén et al., 2012; Seemann et al., 2017; Senju et al., 2011; Stoeber et al., 2012; Yeow et al., 2017).

Caveolin-1, a highly conserved protein of approximately 22 kDa, exists in two isoforms: caveolin-1α and caveolin-1β (Fujimoto et al., 2000). Both isoforms are derived from the CAV1 gene through alternative translation initiation and differ by a short N-terminal segment present in caveolin-1α. Cav1 consists of three main regions: the N-terminal domain, the central hydrophobic domain, and the C-terminal domain (Root et al., 2019; Spisni et al., 2005). The N-terminal domain includes two key regions: the oligomerization domain (residues 61–101) and the scaffolding domain (residues 82–101). The oligomerization domain is required for Cav1 self-oligomerization into homo-oligomers and hetero-oligomerization with caveolin-2, essential for caveolae structural integrity. The caveolin scaffolding domain (CSD) has been proposed to interact with various signaling molecules, including G-proteins, Src family kinases, and endothelial nitric oxide synthase (eNOS) (Couet et al., 1997). The central hydrophobic domain (residues 102–134) is rich in cholesterol-interacting residues and stabilize caveolae within lipid nanodomains of the plasma membrane enriched in cholesterol and sphingolipids. Recent cryo-electron microscopy (cryo-EM) studies reveal that Cav1 assembles into an 8S complex composed of 11 Cav1 protomers arranged in a tightly packed disc with a flat, membrane-embedded surface (Han et al., 2023; Kenworthy, 2023; Ohi and Kenworthy, 2022; Porta et al., 2022). The oligomerization domain, located at the outer rim of the disc, contributes to extensive subunit interactions, while the signature motif forms tight contacts with two neighboring protomers, and the scaffolding domain encircles the periphery of the complex. The C-terminal domain (residues 135–178) remains cytoplasmic and contains multiple palmitoylation sites. Caveolin-1 has been shown to be palmitoylated at three cysteine residues located in the C-terminal domain. However, mutation of these cysteines to serines did not affect proper trafficking of Cav1 to the membrane indicating that palmitoylation may have only a limited impact on the caveolin fold.

### 3.2 Metastable structures at the plasma membrane

Caveolae at the plasma membrane are metastable structures that exist in a dynamic equilibrium, allowing them to rapidly respond to mechanical, biochemical, and environmental stimuli. This metastability is driven by the unique structural organization of caveolin and cavin proteins, interactions with plasma membrane lipids, and their ability to undergo reversible conformational changes in response to cellular environment changes, including mechanical cues and specific signaling pathways (Kenworthy, 2023; Lamaze et al., 2017; Lundmark et al., 2024; Ocket and Matthaeus, 2024; Parton, 2018; Parton et al., 2020; Sinha et al., 2011). In endothelial cells, caveolae respond dynamically to shear stress from blood flow, resulting in Cav1 phosphorylation at Tyr14 and the activation of signaling pathways that promote nitric oxide production, cytoskeletal reorganization, and adaptive cellular responses to maintain vascular homeostasis (Cheng et al., 2015; Rizzo et al., 2003). In adipocytes, caveolae play essential roles in lipid storage and metabolism (Pilch and Liu, 2011).

An essential feature of caveolae is their ability to respond to diverse types of stress including mechanical tension, UV radiation, and oxidative stress. Under increase of membrane tension by osmotic swelling, stretching or shear stress, caveolae rapidly flatten out, buffering membrane tension and preventing cell membrane rupture (Cheng et al., 2015; Sinha et al., 2011). In doing so, caveolae release their coat proteins, in the cytosol, some of which have been reported to affect downstream signaling pathways, thereby attributing a unique role to caveolae as mechanosensors and mechanotransducers (Nassoy and Lamaze, 2012; Parton and del Pozo, 2013). For example, mechanical stress causes EHD2 to detach from the neck of caveolae, undergo SUMOylation, and translocate to the nucleus to regulate gene transcription, including those encoding caveolae components (Torrino et al., 2018). Similarly, UV exposure releases Cavin-3 from caveolae, which interacts with and inhibit PP1α, leading to increased H2AX phosphorylation and apoptosis (McMahon et al., 2019). Caveolins can also exist outside of caveolae as scaffolds (Khater et al., 2019a; Khater, et al., 2019b; Pol et al., 2020). A recent study reveals that caveolae can disassemble into smaller scaffolds under mechanical stress, exposing buried domains like the caveolin scaffolding domain to engage signaling effectors (Mani et al., 2025). Cav1 scaffolds are highly dynamic, rapidly navigating the plasma membrane where they interact with and regulate the activity of key signaling molecules, including JAK1 kinase, PTEN phosphatase, and eNOS.

The metastable nature of caveolae is critical for their diverse cellular roles, including mechanosensing, endocytosis, lipid regulation, and signal transduction. Understanding caveolae metastability offers valuable insights into their functions in health and disease, presenting potential therapeutic opportunities for modulating caveolae dynamics in various pathological conditions.

### 3.3 Caveolin-1 and cancer: a two-faceted conundrum

**FIGURE 2:**
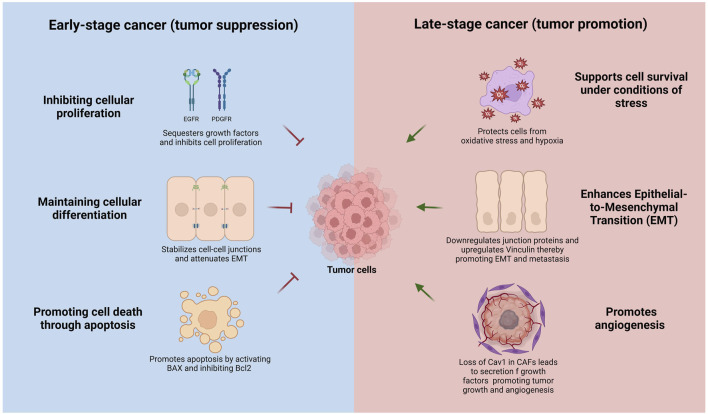
Context-dependent dual roles of Caveolin-1 in cancer progression. In early-stage cancer (left panel, blue), Cav1 acts as a tumor suppressor by inhibiting cellular proliferation through sequestration and negative regulation of growth factor receptors (EGFR, PDGFR), maintaining cellular differentiation by stabilizing adherens and tight junctions, attenuating epithelial-to-mesenchymal transition (EMT), and promoting apoptosis via activation of pro-apoptotic BAX and inhibition of anti-apoptotic Bcl-2. Conversely, in advanced-stage cancer (right panel, red), Cav1 functions as a tumor promoter by supporting cell survival under metabolic stress, oxidative stress, and hypoxia, facilitating EMT through upregulation of mesenchymal markers and downregulation of epithelial junction proteins, enhancing cell migration, and promoting angiogenesis by its loss in cancer-associated fibroblasts (CAFs), which stimulates secretion of pro-tumorigenic growth factors and cytokines.

Caveolin-1 is known to play a complex role in cancer, acting as both a tumor suppressor and promoter depending on the stage of cancer progression (Burgermeister et al., 2008; Chen et al., 2020; Lamaze and Torrino, 2015; Singh and Lamaze, 2020; Williams and Lisanti, 2005). In early-stage cancers, Cav1 often functions as a tumor suppressor by inhibiting cellular proliferation, promoting cell death through apoptosis, and maintaining cellular differentiation (Figure 2) (Quest et al., 2013; Torres et al., 2006; Torres et al., 2007). However, in advanced cancer stages, Cav1 transitions to an oncogenic role and is frequently associated with increased cancer cell survival, drug resistance, and metastatic potential (Shatz and Liscovitch, 2008; van Golen, 2006). Cav1 is often downregulated or lost in breast, colon, ovarian, and lung cancers, suggesting a tumor-suppressive function (Bouras et al., 2004; Hino et al., 2003; Qian et al., 2019; Ren et al., 2021). In contrast, Cav1 is overexpressed in certain aggressive cancers, such as prostate, bladder, liver, and pancreatic cancers, where it plays an oncogenic role (Goetz et al., 2008; Liu et al., 2014; Liu et al., 2016; Raja et al., 2019; Thompson et al., 2010).

The tumor-suppressive role of Cav1 can be mediated through several mechanisms, including the inhibition of proliferative signaling pathways, suppression of oncogenic signaling, activation of pro-apoptotic pathways and senescence, and regulation of metastasis and invasion (Figure 2). Cav1 negatively regulates the activity of several growth factor receptors, such as the epidermal growth factor receptor (EGFR) and platelet-derived growth factor receptor (PDGFR), by binding to these receptors and sequestering them within caveolae (Abulrob et al., 2004; Couet et al., 1997). Recent insights suggest that non-caveolar Cav1 scaffolds can also bind these receptors and inhibit their activity (Lim et al., 2024). Additionally, Cav1 inhibits the Ras-MAPK/ERK pathway and PI3K/AKT pathways, both frequently activated in cancer cells to promote survival and proliferation (Cohen et al., 2004; Engelman et al., 1998; Matthews et al., 2008). Cav1 also impedes epithelial to mesenchymal transition (EMT), a process enabling epithelial cells to acquire invasive and metastatic properties. Cav1 regulates epithelial-to-mesenchymal transition (EMT) by modulating β-catenin–Tcf/Lef-mediated transcriptional pathways. Specifically, Cav1 downregulates the anti-apoptotic protein survivin via inhibition of β-catenin transcriptional activity, a process critically dependent on the presence of E-cadherin. A consequent loss of E-cadherin - a hallmark of EMT -impairs this regulatory mechanism, enhancing cell survival and promoting metastatic potential (Torres et al., 2006; Torres et al., 2007). Cav1 maintains cell-cell adhesion and epithelial integrity by stabilizing adherens and tight junctions, inhibits the expression of transcription factors like Snail, Slug, and Twist, and negatively regulates matrix metalloproteinases (MMPs), involved in extracellular matrix degradation and instrumental to cancer invasion and metastasis (Dalton et al., 2023; Miotti et al., 2005; Nagasaka et al., 2017; Salem et al., 2011; Strippoli et al., 2015).

Conversely, Cav1 can promote cancer progression and metastasis depending on the cellular context by enhancing cell survival under stress, facilitating metastasis and invasion, modulating the tumor microenvironment, and activating pro-oncogenic signaling pathways. Cav1 supports cancer cell survival under metabolic stress, hypoxia, and oxidative stress (Castillo Bennett et al., 2018; Mao et al., 2016). Increased Cav1 expression in certain cancers inhibits pro-apoptotic proteins such as BAX while increasing anti-apoptotic proteins like Bcl-2, thereby promoting cell survival (Zou et al., 2012). In advanced-stage cancers, Cav1 promotes EMT by upregulating mesenchymal markers such as vimentin and N-cadherin while reducing epithelial markers like E-cadherin (Liang et al., 2014; Zhang K. et al., 2016). Furthermore, Cav1 enhances cell migration and invasion by interacting with focal adhesion kinase (FAK) and activating Rho GTPases, which regulate cell motility and cytoskeletal dynamics (Bailey and Liu, 2008). Moreover, Cav1 loss in stromal cells, such as cancer-associated fibroblasts (CAFs), correlates with more aggressive cancer phenotypes, as it promotes the secretion of growth factors and cytokines that drive tumor growth and angiogenesis (Martinez-Outschoorn et al., 2010; Shi et al., 2016; Simpkins et al., 2012; Sotgia et al., 2012; Zhao et al., 2013). Caveolae-mediated mechanosensing regulates the activity of invadosomes, these specialized cellular protrusions that favor cell dissemination through degradation of collagen fibers (Monteiro et al., 2023).

### 3.4 Caveolin-1 as a secreted protein

Caveolin-1 is traditionally described as a membrane-associated protein, crucial for the formation of caveolae and the regulation of various signaling pathways. However, studies have revealed that Cav1 can also be found as a secreted protein, functioning outside the cell to influence intercellular communication, inflammation, cancer progression, and tissue repair. The discovery of Cav1 as a secreted protein dates back to 1999, when Liu et al. reported Cav1 sequestration in cytoplasmic lipoprotein vesicles in mouse pancreatic exocrine cells. In this study, pancreases from mice treated with different secretagogue mixtures were removed and cultured in media. The presence of Cav1 in the culture media provided the first evidence of Cav1 secretion outside cells (Liu et al., 1999). It is now understood that Cav1 can be secreted via non-classical pathways, including EV-mediated secretion.

## 4 Caveolin-1 in EV biogenesis

Cargo sorting is a critical step during EV biogenesis. This process is tightly regulated by diverse molecular mechanisms that coordinate the incorporation of proteins, lipids, metabolites, and genetic material into EVs.

Cav1 is integral to the formation of membrane curvature, a critical aspect of EV biogenesis. As a principal component of caveolae, Cav1 facilitates membrane curvature through its oligomerization and interaction with cholesterol- and phosphatidylserine-enriched lipid nanodomains at the plasma membrane (Doktorova et al., 2025; Zhou et al., 2021). This structural role of Cav1 in membrane deformation suggests a potential mechanism by which it may influence EV biogenesis. In addition, the interaction between Cav1 and cholesterol has been associated with regulation of exosome formation and cargo sorting in MVBs. It was demonstrated that Cav1 regulates exosome biogenesis and exosomal protein cargo sorting by controlling cholesterol levels at the MVBs, acting as a “cholesterol rheostat (Albacete-Albacete et al., 2020).

While direct evidence linking Cav1-induced membrane curvature to EV budding is still under investigation, the established functions of Cav1 in membrane dynamics and curvature support its possible involvement in EV formation processes (Table 1).

**TABLE 1:** Cav1 partners in EV biogenesis.

Partner molecules	Model	Description	References
PS, cholesterol	MCF7, MDCK	Cav1 interaction with lipids regulates membrane curvature and lipid nanodomain formation, suggesting an essential role in EV biogenesis	Zhou et al. (2021)
Cholesterol	MEF	Cav1 regulates cholesterol content in MVBs, regulating exosome biogenesis	Albacete-Albacete et al. (2020)
ESCRT-0	Various cancer cells	Along with Cav1 is responsible for EV secretion after mechanical stress	Saquel et al. (2025) (Biorxiv)
hnRNPA2B1, hnRNPA1, hnRNPK	Various cancer cells	RNA-binding proteins involved in miRNA sorting into EVs. Cav1 guides them towards MVBs for loading	Lee et al. (2019), Li et al. (2021), Robinson et al. (2021)
Argonaute2	Various cancer cells	Ago2-Cav1 interaction enhances the release of miRNAs via EVs	Lin et al. (2024)
Tenascin-C, Cyr61, S100A9	MEF, various cancer cells	ECM and ECM-associated proteins enriched in EVs in the presence of Cav1	Albacete-Albacete et al. (2020), Campos et al. (2018), Campos et al. (2023); Saquel et al. (2025) (Biorxiv)
Fas/Fap1	MSCs, mouse	Forms complex with Cav1 to increase release of IL-1RA through EVs to promote wound healing	Kou et al. (2018)
Cavin1	Glioblastoma, mouse	Regulates caveolae formation and EV release	Hong et al. (2024)

Recent findings from our group suggest that mechanical stress induces a significant increase of small EV release in cancer cells. This release was found to be Cav1 and ESCRT-0-dependent, indicating that Cav1 plays a crucial role in the mechanosensing pathways that govern EV biogenesis under physical stress conditions (Saquel et al., 2025). Furthermore, these Cav1-depentent EVs released after mechanical stress show a preferential tropism towards the liver.

### 4.1 EV cargo and biomarker potential

Cav1 is commonly found in EVs from various cell types. Notably, Cav1 is abundant in TEVs and has been proposed as a biomarker for cancer progression (Campos et al., 2019). In melanoma, EVs purified from human plasma samples revealed significantly high levels of Cav1/Rab-5b double-positive EVs in patients compared to healthy donors, underscoring its potential as a disease biomarker (Logozzi et al., 2009). Further supporting this notion, proteomic analyses of exosomes derived from melanoma cell lines, with varying degrees of aggressiveness, revealed a distinct protein signature associated with metastatic cell lines. This signature included oncoproteins involved in cellular migration, angiogenesis, and immune responses, with Cav1 being a central component (Lazar et al., 2015). In a broader cancer context, proteomic profiling of EVs from the NCI-60 panel, comprising 60 human cancer cell lines from cancerous tissues such as the brain, colon, breast, kidney, prostate, and ovary, identified Cav1 as a common protein cargo across multiple cancer types, alongside other cancer-specific biomarkers (Hurwitz et al., 2016). Beyond being a significant cargo protein, Cav1 also plays an active role in selecting and recruiting specific miRNAs and proteins for EV incorporation (Albacete-Albacete et al., 2020; Campos et al., 2018; Campos et al., 2023).

### 4.2 miRNA and protein sorting

The selective loading of miRNAs into EVs involves RNA-binding proteins, primarily heterogeneous nuclear ribonucleoproteins (hnRNPs). These proteins recognize and bind to distinct nucleotide sequences in miRNAs, guiding their incorporation into EVs. The strength and specificity of the interaction between hnRNPs and miRNAs plays a crucial role in determining which miRNAs are preferentially sorted into EVs. Additionally, post-translational modifications of hnRNPs, such as sumoylation, phosphorylation or methylation, among others, can influence their biological functions, including their binding preferences, thereby modulating the miRNAs sorting process (Villarroya-beltri et al., 2013).

Cav1 is essential for sorting specific miRNAs into EVs. Phosphorylation of Cav1 at tyrosine 14 (pY14) promotes its interaction with O-GlcNAcylated hnRNPA2B1, inducing the trafficking of the Cav1/hnRNPA2B1 complex into EVs together with specific miRNA subsets (Lee et al., 2019). Additionally, Cav1 interacts with SUMOylated hnRNPA1, assisting in miRNA loading into EVs. The absence of Cav1 reduces hnRNPA1 levels in EVs, impairing their ability to promote tumor proliferation and migration (Li et al., 2021). Another microRNA-binding protein, hnRNPK, is also regulated by Cav1. In the absence of cavin-1, non-caveolar Cav1 guides hnRNPK into MVBs, facilitating miRNA recruitment for exosomal release. Membrane lipid composition, such as cholesterol depletion, modulates this process. Importantly, hnRNPK has been linked to bone metastasis, as its knockdown in prostate cancer cells impairs EV-induced osteoclastogenesis. Elevated hnRNPK levels have also been detected in biofluid EVs from metastatic cancers (Robinson et al., 2021).

The role of Cav1 in cargo sorting extends beyond miRNA sorting to include specific proteins. Cav1 mediates the selective incorporation of ECM proteins, such as Tenascin-C, CYR61, and fibronectin, into EVs. These proteins are crucial for cellular adhesion and migration (Albacete-Albacete et al., 2020; Campos et al., 2018; Campos et al., 2023). However, the precise molecular mechanisms by which Cav1 directs protein sorting into EVs remains unclear (Table 1).

### 4.3 Pro-tumorigenic cargo

The presence of Cav1 in TEVs not only serves as a potential biomarker for cancer progression but also actively promotes malignancy by facilitating the acquisition of cancerous traits in recipient cells. Cav1 expression is notably elevated in melanoma cell lines, and its silencing has been shown to reduce tumor growth and angiogenesis. Cav1 bearing EVs stimulate anchorage independence, migration, and invasion through paracrine and autocrine mechanisms (Felicetti et al., 2009). These EVs can transfer metastatic properties from highly aggressive melanoma cells to less aggressive recipient cells. Proteomic analyses of EVs from various melanoma cell lines have revealed that their protein composition varies with the aggressiveness of the parental cells, with Cav1 levels in EVs specifically correlating with the metastatic potential of the corresponding cell lines (Lazar et al., 2015).

Low extracellular pH, a hallmark of many tumors, enhances pro-cancerous traits such as invasion, migration, and proliferation (Corbet and Feron, 2017). In melanoma cells, acidic microenvironments increase EV release from donor cells and facilitates Cav1 delivery to recipient cells (Parolini et al., 2009). In hepatocellular carcinoma (HCC), TEVs enriched with Cav1 and Cav2 have been shown to induce migration and invasion in non-motile hepatocytes. These EVs, which are secreted predominantly by highly metastatic HCC cell lines (MHCC97L and HKCI-8), highlight Cav1’s role in selectively packaging proteins and RNAs associated with metastatic behavior (He et al., 2015).

In metastatic breast cancer, Cav1-loaded EVs released by cancer cells, have been shown to confer pro-metastatic traits, such as enhanced invasion and migration to non-metastatic recipient cells. This effect is partly attributed to Cav1’s role in sorting specific ECM proteins into EVs, which facilitates local tumor microenvironment remodeling and stromal niche formation in distant tissues (Albacete-Albacete et al., 2020; Campos et al., 2018; Campos et al., 2023).

Tumor progression shares many features with wound healing. In this regard, a Fas/Fap-1/Cav1 complex has been identified as a key regulator of IL-1RA-enriched EV secretion in mesenchymal stem cells. This study demonstrated that Cav1 acts as a crucial scaffold protein within this complex, facilitating SNARE-mediated membrane fusion, which is essential for the release of small, anti-inflammatory EVs, particularly under TNF-α stimulation, which enhances wound healing in mice (Kou et al., 2018).

Beyond promoting cancerous traits, Cav1 modulates EV dynamics to support cancer progression. In breast cancer, Cav1 interacts through its scaffolding domain CSD with argonaute-2 (Ago2), a key player in RNA-mediated gene silencing. The Cav1-Ago2 interaction regulates miRNA-mediated mRNA suppression and enhances the release of miRNAs via EVs, contributing to metastasis and chemoresistance (Lin et al., 2024). Additionally, TRAF4, a scaffold protein with E3 ubiquitin ligase activity, binds to Cav1 and stabilizes it by preventing its ubiquitin-dependent degradation, thereby activating pro-tumorigenic signaling pathways. Disruption of this interaction reversed chemoresistance to temozolomide in glioblastoma (Li et al., 2022). In glioblastoma cells, Cav1’s interaction with Cavin-1 is also essential for EV secretion and temolozomide efflux, as disrupting this interaction reduces EV secretion, increases intracellular drug retention, and enhances drug sensitivity (Hong et al., 2024). Collectively, these findings highlight Cav1’s multifaceted role in cancer drug resistance and EV dynamics (Table 1).

### 4.4 EV uptake

Cav1 is as a key regulator of PM lipid nanodomains, orchestrating the recruitment of specific lipid species such as cholesterol, phosphatidylserine, and sphingomyelin, to create distinct lipidic environments (Prakash et al., 2021; Sonnino and Prinetti, 2009). These lipid nanodomains act as platforms for clustering transmembrane proteins and proteins with cholesterol or lipid feature affinities, such as specific acyl chains (Harayama and Antonny, 2023; Sezgin et al., 2017). This clustering mechanism suggests that Cav1 may facilitate the preferential assembly of surface molecules on recipient cells, enhancing interactions with EVs and influencing docking, fusion, and clathrin-independent endocytosis, ultimately impacting EV uptake efficiency.

However, EV uptake dynamics are complex and not exclusively dependent on PM lipid composition. Studies indicate that Cav1 inhibition reduces EV uptake in various cell lines, including lung cancer cells (Javeed et al., 2015; Nanbo et al., 2013; Wei et al., 2017). Conversely, Cav1 upregulation in hippocampal neurons under ischemic conditions enhances EV uptake, acting as a neuroprotective mechanism against apoptosis (Yue et al., 2019). Intriguingly, some evidence suggests that Cav1 or caveolae might also hinder EV uptake by modulating signaling pathways such as ERK1/2, where Cav1 downregulation correlates with increased uptake (Svensson et al., 2013).

These conflicting observations highlight the complexity and context-dependent role of Cav1 in EV dynamics and uptake. The interpretation of these findings is further complicated by the frequent use of pharmacological agents (e.g., filipin, dynasore, nystatin) that lack specificity for Cav1 and can disrupt overall membrane integrity, thereby confounding experimental outcomes. Moreover, the co-regulation of Cav1 and cavin1 transcription suggests broader effects on membrane lipid composition, which may directly influence EV uptake via changes in membrane structure and dynamics (Hill et al., 2008).

## 5 Conclusion

The intricate role of Cav1 in EV dynamics represents a fertile ground for advancing our understanding of cellular communication in cancer. Cav1 has emerged as a key regulator of EV biogenesis, cargo sorting, and uptake, with profound implications for cancer progression, metastasis, and therapeutic resistance. Despite significant advancements, many aspects of Cav1 function in EV biology remain unclear or controversial, highlighting the need for further investigation.

Cav-1-positive EVs play a pivotal role in intercellular communication within the TME, facilitating the transfer of oncogenic signals, promoting drug resistance, and enhancing tumor aggressiveness. Given the ability of EVs to travel long distances, Cav1-positive EVs may also play a role in pre-metastatic niche formation, priming distant tissues for colonization by cancer cells. Targeting Cav-1 in cancer cells and their EVs offers promising therapeutic opportunities, including inhibiting tumor growth, preventing metastasis, overcoming drug resistance, and improving overall patient outcomes.

The dual nature of Cav1, acting as both an oncogene and tumor suppressor depending on cancer type and stage, adds complexity to its therapeutic targeting. In cancers where Cav1 is downregulated, restoring its expression could increase sensitivity to chemotherapy and radiation therapy, inhibit pro-survival pathways (e.g., PI3K/AKT), and improve the efficacy of targeted therapies like EGFR inhibitors. Additionally, Cav1 restoration may enhance immunotherapies by modulating immune cell behavior and boosting cytotoxic T-cell or natural killer cell activity.

Conversely, in cancers where Cav-1 is upregulated, inhibiting Cav1 could suppress tumor proliferation, invasiveness, and angiogenesis. Cav1’s role in stromal crosstalk suggests that its inhibition could disrupt the supportive tumor niche by reducing fibroblast activation, angiogenesis and immune evasion. Downregulating Cav1 may also enhance the immune response by activating macrophages and dendritic cells and reducing immunosuppressive mechanisms, such as regulatory T-cell recruitment and expression of immune checkpoint molecules such as PD-L1.

Future therapeutic strategies for Cav1 modulation include small molecules, peptides, gene therapies, RNA-based approaches, and nanoparticles or antibody-drug conjugates delivery systems. Small molecule inhibitors or peptides could selectively inhibit or enhance Cav-1 function depending on its context-specific role. Gene therapy, using viral vectors or CRISPR/Cas9-based systems, may restore Cav-1 expression in tumor-suppressive contexts, while RNA-based therapies (e.g., siRNA or miRNA) could inhibit Cav-1 expression in cancers where it functions as an oncogene. Existing drugs that indirectly modulate Cav1 activity, such as statins, also hold promise for repurposing in combination therapies.

Harnessing advanced genomic, proteomic, and bioinformatic tools will be essential for identifying precise therapeutic windows for Cav1 modulation and minimizing off-target effects. Given the context-dependent roles of Cav1, refining these approaches through preclinical and clinical research will be crucial to ensure their safety and efficacy. Looking ahead, Cav1 holds significant promises as both a biomarker and therapeutic target. Integrating Cav1-based strategies into clinical practice could be a game changer in cancer diagnosis, prognosis, and treatment, ultimately improving patient outcomes and advancing the field of precision oncology.
